# Short-Term Responses of Soil Microbial Communities to Changes in Air Temperature, Soil Moisture and UV Radiation

**DOI:** 10.3390/genes13050850

**Published:** 2022-05-10

**Authors:** Isabel Silva, Marta Alves, Catarina Malheiro, Ana Rita R. Silva, Susana Loureiro, Isabel Henriques, M. Nazaret González-Alcaraz

**Affiliations:** 1 CEF (Center for Functional Ecology), Department of Life Sciences, Faculty of Sciences and Technology, University of Coimbra, 3000-456 Coimbra, Portugal; silva.isabel@ua.pt; 2 CESAM (Centre for Marine and Environmental Studies), Department of Biology, University of Aveiro, 3810-193 Aveiro, Portugal; catarinalemosmalheiro@ua.pt (C.M.); ritas@ua.pt (A.R.R.S.); sloureiro@ua.pt (S.L.); 3 CBQF—Center for Biotechnology and Fine Chemistry, School of Biotechnology, Portuguese Catholic University, 4169-005 Porto, Portugal; msalves@ucp.pt; 4 Department of Agricultural Engineering of the E.T.S.I.A. & Soil Ecology and Biotechnology Unit of the Institute of Plant Biotechnology, Technical University of Cartagena, 30203 Cartagena, Spain

**Keywords:** increased temperature, drought, flood, UV exposure, microbial activity, bacterial diversity, metagenomics, quantitative PCR, soil microbiome, soil invertebrates

## Abstract

We analyzed the effects on a soil microbial community of short-term alterations in air temperature, soil moisture and ultraviolet radiation and assessed the role of invertebrates (species *Enchytraeus crypticus*) in modulating the community’s response to these factors. The reference soil, Lufa 2.2, was incubated for 48 h, with and without invertebrates, under the following conditions: standard (20 °C + 50% water holding capacity (WHC)); increased air temperature (15–25 °C or 20–30 °C + 50% WHC); flood (20 °C + 75% WHC); drought (20 °C + 25% WHC); and ultraviolet radiation (UV) (20 °C + 50% WHC + UV). BIOLOG EcoPlates and 16S rDNA sequencing (Illumina) were used to assess the microbial community’s physiological profile and the bacterial community’s structure, respectively. The bacterial abundance (estimated by 16S rDNA qPCR) did not change. Most of the conditions led to an increase in microbial activity and a decrease in diversity. The structure of the bacterial community was particularly affected by higher air temperatures (20–30 °C, without *E. crypticus*) and floods (with *E. crypticus*). Effects were observed at the class, genera and OTU levels. The presence of invertebrates mostly resulted in the attenuation of the observed effects, highlighting the importance of considering microbiome–invertebrate interactions. Considering future climate changes, the effects described here raise concern. This study provides fundamental knowledge to develop effective strategies to mitigate these negative outcomes. However, long-term studies integrating biotic and abiotic factors are needed.

## 1. Introduction

Soil is a non-renewable resource that is currently being depleted at a fast rate. Climate change plays an important role in soil degradation. Alterations in air temperature and soil moisture content, for example, can lead to changes in soil organic and mineral components that may, consequently, alter soil structures and result in soil degradation [1].

Soil microorganisms, collectively referred to as the soil microbiome, are key factors in the preservation of soil health [2,3]. The soil microbiome is responsible for crucial functions and services for terrestrial ecosystems, such as by contributing to the cycling of organic matter and nutrients [4]. The soil microbiome could also contribute to the mitigation of the negative effects of climate change on soils [5]. When used as part of a strategy to mitigate the effects of droughts, for example, microorganisms can produce polymeric substances that plug the pores of soils to improve water retention [5]. Furthermore, through the microbial uptake of carbon exported from plant roots, microorganisms can increase soil carbon and, consequently, soil health [5,6].

The acceleration in the rate of climate alterations [7] could jeopardize the crucial and beneficial functions performed by soil microorganisms. In fact, McHugh et al. [8] concluded that instead of directly affecting soil properties, changes in climate conditions play a larger role in altering the structure of soil microbial communities. For example, flood situations were shown to create anaerobic soil areas, due to water saturation, which led to relevant shifts in soil community structure [9]. Similarly, in a 26-year soil warming experiment, Melillo et al. [10] found reductions in soil microbial biomass and changes in community composition. Considering the critical role the soil microbiome plays in numerous functions, changes in this community might result in serious consequences for terrestrial ecosystems. It is known, for instance, that alterations in the microbiome’s diversity decrease its multifunctionality, i.e., its ability to maintain multiple ecosystem functions and services simultaneously [11]. Furthermore, if functionally important taxa disappear from soil communities, substantial effects are expected, for example, in soil fertility. These changes might, in turn, be reflected in above-ground diversity and productivity. In extreme scenarios, these changes can lead to soil desertification, with negative environmental, social and economic effects as a consequence [12].

Understanding the effects of climate change on the soil microbiome is therefore crucial to preserve soil ecosystem functions and services. However, it is still unclear how microorganisms respond to climate alterations. This response is difficult to predict and is expected to depend on the type of soil ecosystems. Reasonably, the effects of temperature alterations on soil microorganisms are different in the Arctic, in wetlands and in drylands [5]. Furthermore, the interaction of the soil microbiome with other biotic components of soils, such as invertebrates, might influence the soil microbiome’ response to climate alterations. Thus, to forecast future changes in ecosystem functioning due to climate alterations and to preserve the key roles of the soil microbiome in ecosystems, a better understanding of soil microbiome responses to climate change is needed. 

Although the effects of climate change cannot simply be inferred from short-term studies, such studies can provide important insights into the responses of communities to extreme and occasional events and the extent to which these responses are influenced by other soil organisms. Considering all this, our work aimed to study the responses of the microbial community of a reference soil to short-term alterations in air temperature, soil moisture content and ultraviolet (UV) radiation. Moreover, we aimed to assess whether the response of the soil microbial community to the tested conditions could have been influenced by the presence of soil invertebrates (soft-bodied oligochaete species *E. crypticus*). We hypothesized that short-term exposure to alterations in air temperature, soil moisture content and UV radiation induces rapid changes in the activity, structure, composition and functions of the soil microbial community and, further, that these changes depend on the modulated scenario and on the presence or absence of the studied invertebrate. To test these hypotheses, the reference soil, Lufa 2.2, a natural soil from the German Agricultural Investigation and Research Speyer Institute (LUFA Speyer), was incubated for a short period of time under different scenarios that mimicked rising air temperature, drought or flood situations and increasing exposure to UV radiation; we then compared its responses to its responses under standardized conditions. The scenarios tested were selected based on the Intergovernmental Panel on Climate Change (IPCC)’s projections of future climate alterations in Europe by 2100 [13,14,15]. Soil incubations were carried out without and with the presence of the soil invertebrate *E. crypticus*. This invertebrate species was selected due to the key role it plays in terrestrial ecosystems by participating in the biogeochemical cycling of organic matter and nutrients and its improvement of soil structures [16,17]. After exposure, the changes in microbial catabolic activity and in the structure and functions of the soil microbial community were evaluated. 

## 2. Materials and Methods

### 2.1. Standard Reference Soil

The standard reference soil, Lufa 2.2, was purchased from the Agricultural Investigation and Research Institute (LUFA) in Speyer, Germany. This non-contaminated natural soil is widely used in environmental studies to evaluate the impact of different types of stressors (chemical and natural) on soil compartments [18,19,20]. The soil is characterized by a sandy, loamy texture (≈76–86% sand, ≈10–16% silt and ≈4–8% clay), pH in 0.01 M CaCl_2_ ≈ 5.4–5.6, organic carbon (OC) ≈ 1.6–1.8%, nitrogen (N) ≈ 0.17–0.19% and cation exchange capacity (CEC) ≈ 9.2–9.8 cmol_c_ kg^−1^ (http://www.lufa-speyer.de, accessed on 8 April 2020). The water holding capacity (WHC) of the soil was determined in porous-base glass cylinders after soil saturation with water for 3 h followed by 2 h of excess-water removal [21]; WHC ≈ 44–46%.

### 2.2. Soil Invertebrates

The soil invertebrate species *E. crypticus* (phylum Annelida, class Oligochaeta, family Enchytraeidae) was cultured in agar medium prepared with aqueous Lufa 2.2 soil extracts and maintained at 20 °C in complete darkness. Cultures were fed weekly with a mixture of oatmeal, dry yeast, milk, yolk powder and fish oil. Sexually mature individuals (clearly visible clitellum) of ≈1 cm in length and without visible problems were used for the performance of soil incubations. *E. crypticus* shows a broad tolerance range to distinct soil properties, including pH (3.6–8.2), organic matter content (1.2–42%) and clay particles (1–29%) [22,23,24].

### 2.3. Experimental Soil Incubations

The standardized OECD guidelines recommend evaluating the C and N transformation activities of soil microorganisms at a constant air temperature of 20 °C and a soil moisture content of ≈50% maximum WHC, without controlling UV radiation exposure (these are the standard conditions referred to hereafter) [25,26]. From this point and considering the IPCC projections for the end of the 21st century [13,14,15], a set of scenarios was established to evaluate the effects of air temperature, soil moisture content and UV radiation on the soil microbial community, comparing the response under each of the scenarios simulated with respect to the standard conditions. The different scenarios established were simulated by varying one single factor (air temperature, soil moisture content or UV radiation) and keeping the remaining factors at the levels recommended by the OECD guidelines. The incubation time for testing each scenario was 48 h. Table S1 (Supplementary Material) summarizes the different scenarios simulated for each of the factors evaluated. 

Two scenarios were established for air temperature considering the rising air temperature predictions. The air temperature scenarios simulated consisted of repetitive air temperature regimes 24 h in duration and with a thermal amplitude of 10 °C (minimum–maximum air temperature reached): (i) 15–25 °C; (ii) 20–30 °C. Each regime cycle started from the minimum air temperature (15 °C and 20 °C, respectively) at 8:00 a.m. and gradually increased until 12:00 a.m., when the maximum air temperature was reached (25 °C and 30 °C, respectively); the cycles were maintained until 4:00 p.m. From this time onward, the air temperature gradually decreased until 4:00 a.m., when the minimum air temperature was reached again and maintained until 8:00 a.m. to start a new air temperature regime cycle. The air temperature scenarios simulated are graphically represented in Figure S1a, Supplementary Material. Two scenarios were established for soil moisture content considering the predicted increased frequency and intensity of extreme weather events (Figure S1b, Supplementary Material): (i) soil moistened at 75% maximum WHC to simulate greater soil water availability after intense rainfall events and/or floods (hereafter flood conditions); (ii) soil moistened at 25% maximum WHC to simulate lower soil water availability during severe dry spells (hereafter drought conditions). One scenario was established with respect to UV radiation (Figures S1c and S2, Supplementary Material). It consisted of a repetitive UV radiation regime 24 h in duration (6 h of UV radiation emission and 18 h without UV radiation) and a total daily UV dose of 4400 J m^−2^. Two UV indexes were defined for the UV radiation period according to the following UV radiation exposure categories: 7 (high UV intensity) and 10 (very high UV intensity). The regime started without UV radiation emission at 8:00 a.m. and lasted until 10:00 a.m. From this point, UV radiation corresponding to an UV index of 7 emitted until 12:00 a.m., when it intensified to 10. The UV index of 10 was maintained until 2:00 p.m., when it decreased again to 7 until 4:00 p.m. From this time onward, the UV radiation emission ceased until 8:00 a.m., when a new cycle of UV radiation regime began. The UV radiation scenario simulated is graphically represented in Figure S1c in the Supplementary Material. More details on the simulated UV radiation scenario are available in the Supplementary Material.

Soil incubations were performed in 170-milliliter glass jars (9.5 cm high and 5 cm in diameter) containing 50 g of soil previously watered at the moisture content to be tested (five replicates). Jars with soil invertebrates were also prepared (30 *E. crypticus* individuals per jar; 5 replicates). Test jars were covered with perforated parafilm and incubated for 48 h in acclimatized chambers/rooms with a 16:8 h light:dark photoperiod under standard conditions and different air temperature, soil moisture content and UV radiation scenarios established (Table S1, Supplementary Material). Air temperature incubations were performed in a KBWF 720 Binder acclimatized chamber (Binder, Germany). Soil moisture and UV radiation incubations were carried out in acclimatized rooms. After the incubation period, soils were sampled for microbiological analysis: (i) fresh soil samples were used for community-level physiological profile determination; (ii) soil samples were frozen with liquid N and stored at −80 °C for DNA extraction. In the case of test jars containing soil invertebrates, caution was taken not to sample *E. crypticus* individuals.

### 2.4. Community Level Physiological Profiles

After 48 h of exposure to the different scenarios established, community-level physiological profiles (CLPPs) were determined in all replicates using the Biolog EcoPlate system (BIOLOG Inc., Biolog, Hayward, CA, USA), according to Samarajeewa et al. [27]. Briefly, 3 g of fresh soil was shaken at 200 rpm in 27 mL of sterile water with 20 sterile glass beads, for 10 min, at room temperature. Soil suspensions were then diluted to obtain a 100-fold working solution. A total of 100 µL of the working solution was then inoculated into each well and EcoPlates were incubated at 20 °C in the dark for 6 d. The optical densities of each well were measured at 590 nm every 24 h using an automated plate reader (Biolog, MicroStation, Hayward, CA, USA). Microbial catabolic activity was calculated as the average well color development (AWCD), as previously described by Samarajeewa et al. [27]. Preference for C-source group consumption was categorized as the substrate average well color development (SAWCD). Substrates were classified within five C-source groups according to Frąc et al. [28]: polymers; carbohydrates; carboxylic and acetic acids; amino acids; amines and amides.

### 2.5. DNA Extraction and Quantitative PCR

Total genomic DNA was extracted after 48 h of exposure to the different scenarios established (0.25 g of soil; three replicates per condition) using a PureLink microbiome DNA purification kit (Invitrogen, Thermo Fisher Scientific, Carlsbad, CA, USA), following manufacturer’s instructions.

Quantitative PCR (qPCR) was used to determine total bacterial abundance. The quantification of the 16S rRNA gene was carried from each DNA sample in three independent DNA reactions with primers 338F (CCTACGGGAGGCAGCAG) and 518R (CCTACGGGAGGCAGCAG) [29]. The amplification reaction mixture (final volume of 20 μL) contained 2 μL of the DNA template, 0.4 μL of each primer solution at 10 µM, 10 µL of NZYSpeedy qPCR Green Master Mix (3 mM MgCl_2_) (Nzytech, Portugal) and 7.2 μL of ultra-pure water. PCR reactions were performed using a CFX96™ real-time PCR system (Bio-Rad, Hercules, CA, USA) under the following thermal conditions: 95 °C for 3 min, followed by 30 amplification cycles consisting of 95 °C for 15 s and 65 °C for 30 s. Melting analysis was performed from 55 °C to 95 °C, with steady 0.1 °C increments at each 5 s. Quantification was performed based on the standard curve method, as described by Brankatschk et al. [30].

### 2.6. Illumina High-Throughput Sequencing

The 16S rRNA gene Microbiome Profiling with MiSeq was performed by Eurofins Genomics (Ebersberg, Germany) by amplifying the hypervariable V3–V4 region. The resulting data were analyzed as follows, according to Eurofins Genomics standard protocols: demultiplexing, read merging using FLASH software (2.2.00, [31]), quality filtering (to remove reads with less than 285 bp, ambiguous bases (“N”) and sequences with an average quality lower than Q30), identification and removal of chimeric reads using UCHIME algorithm in VSEARCH software package [32]; operational taxonomic unit (OTU)-picking using minimum entropy decomposition (MED) [33]; and assignment of taxonomic information to each OTU. OTU assignment was performed by DC-MEGABLAST alignments of representative cluster sequences to the NCBI database. A sequence identity of 70% across at least 80% of the representative sequence was a minimal requirement for considering reference sequences [34]. Abundance of bacterial taxonomic units was normalized using lineage-specific copy numbers of the marker genes to improve estimates [35]. Nucleotide sequences were deposited in GenBank under the accession numbers SAMN20256386 to SAMN20256421. 

### 2.7. Inference of Soil Microbiome Functions from 16S rRNA Gene Sequencing Data

Based on the OTU abundance table, Piphillin software [36] was used to predict the effects of the different scenarios tested on soil microbiome functions. OTUs were matched to Kyoto Encyclopedia of Genes and Genomes (KEGG; http://www.genome.jp/kegg/, accessed on 15 August 2019) database of phylogenetically referenced prokaryotic genomes using an identity cut-off of 97% to obtain a list of KEGG orthologs (KO) and their abundance.

### 2.8. Statistical Analysis

Statistical analyses were performed using IBM SPSS Statistics v26 (IBM Corporation, Endicott, NY, USA). A significance level of 0.05 was considered. Normal distribution of the data and equal variance were verified by the Shapiro–Wilk test and Levene’s test, respectively. One-way ANOVA followed by Dunnett’s post hoc test was performed to check for differences between the standard conditions (i.e., conditions recommended by standardized OECD guidelines) and each of the scenarios simulated (rising air temperature, drought or flood situations and increasing exposure to UV radiation). This was performed separately for the soil incubations without and with invertebrates’ presence. 

The α and β diversity metrics were calculated based on OTU abundance table. Species richness (number of observed OTUs) and diversity (Shannon–Wiener index) were obtained through vegan package [37] of R software v.4.0.2 [38]. PRIMER v6 (Primer-E Ltd., Plymouth, UK) was used to perform cluster and principal coordinate analysis (PCoA) using a Bray–Curtis distance matrix.

## 3. Results

### 3.1. Community Level Physiological Profiles

**Figure 1:**
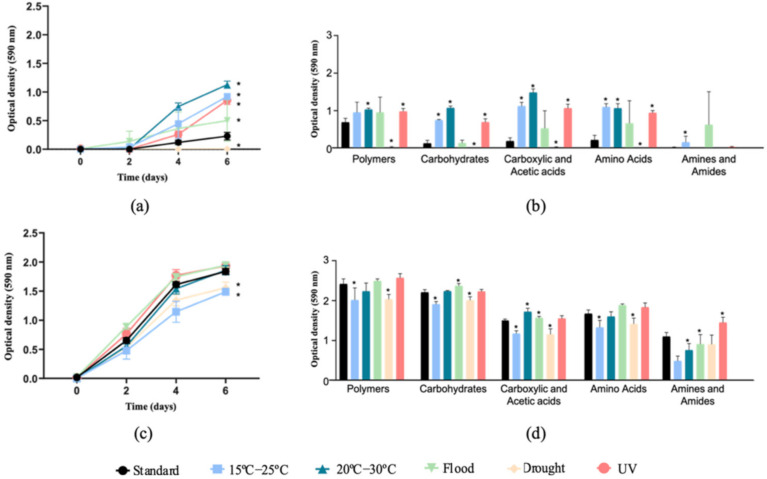
Community-level physiological profiles based on average well color development (AWCD, **a**,**c**) and substrate average well color development (SAWCD, **b**,**d**) of Lufa 2.2 soil without (**a**,**b**) and with (**c**,**d**) the presence of the soil invertebrate *E. crypticus*. After exposure to the different scenarios for 48 h, microbial catabolic activity was evaluated for 6 days. (**b**,**d**) show substrate consumption at the end of the 6 days. Values are average ± standard deviation (*n* = 5). Standard refers to the conditions recommended by the standardized OECD guidelines. The symbol * indicates statistical differences compared to standard conditions based on one-way ANOVA followed by Dunnett’s post hoc test (*p* < 0.05). UV (ultraviolet).

In the soils without *E. crypticus*, an increase in microbial catabolic activity was observed in relation to the standard conditions after exposure to all the scenarios simulated, except for drought (Figure 1a). The highest substrate consumption rates were found for rising air temperature (300% and 390% increase in AWCD under 15–25 °C and 20–30 °C, respectively) and UV radiation (270% increase in AWCD). Specifically, a higher oxidation of polymers (38%–50%–42%, for the three scenarios mentioned above, respectively), carbohydrates (484%–742%–443%), carboxylic and acetic acids (501%–695%–472%) and amino acids (406%–390%–334%) was observed (Figure 1b). The amine and amide oxidation increased by more than 1000% after exposure to rising air temperatures ranging between 15 °C and 25 °C. Regarding the flood condition, no significant differences were observed in the microbial consumption of the substrates tested after the exposure to this scenario. The opposite effects were observed when the soil was exposed to drought conditions, which led to a decrease in AWCD of 97% compared to the standard conditions (with almost no oxidation of any of the carbon sources tested) (Figure 1a,b).

The presence of *E. crypticus* in the soil stimulated microbial catabolic activity (699% increase in AWCD under standard conditions) (Figure 1c). Furthermore, the results showed that the presence of the soil invertebrates attenuated the effects of the different scenarios tested on the soil microbial communities’ activity. A decrease in AWCD was observed after soil exposure to air temperatures ranging between 15 °C and 25 °C (a decrease of 19% in relation to standard conditions) and drought conditions (a 15% decrease) (Figure 1c). After the exposure to temperatures between 15–25 °C and drought, a lower utilization of all carbon sources tested was observed, except for the amines and amides (17%–16% of polymers, 14%–9% of carbohydrates, 22%–23% of carboxylic and acetic acids and 20%–15% of amino acids, respectively) (Figure 1d). Conversely, compared to the drought scenario, the flood condition resulted in a higher oxidation of carbohydrates (8%) and carboxylic and acetic acids (5%) (Figure 1d).

### 3.2. Bacterial Community Structure and Composition

#### 3.2.1. Bacterial Abundance and α and β-diversity

Compared to standard conditions, no significant differences were observed in bacterial abundance after exposure to all the scenarios simulated (Figure S3, Supplementary Material). The bacterial absolute abundances (16S rRNA gene copy number/g soil) varied between 2.35 × 10^9^ ± 2.22 × 10^9^ (flood) and 8.11 × 10^9^ ± 2.32 × 10^9^ (15–25 °C) in the soil without invertebrates and between 3.10 × 10^8^ ± 1.83 × 10^7^ (20–30 °C) and 8.01 × 10^8^ ± 3.89 × 10^8^ (drought) in the soil with invertebrates. The presence of the *E. crypticus* in the soil did not change the response of the bacterial communities to the different scenarios tested.

**Figure 2:**
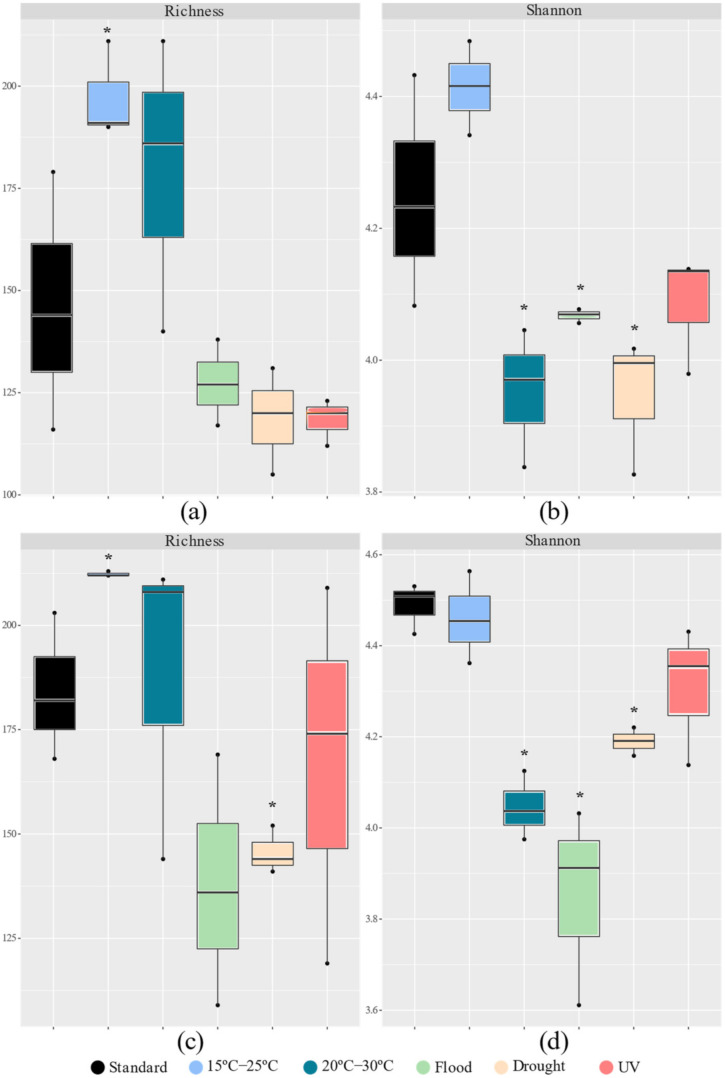
The α diversity measures (richness **a**,**c**; and Shannon index **b**,**d**) based on 16S rRNA gene profiling of Lufa 2.2 soil without (**a**,**b**) and with (**c**,**d**) the presence of the soil invertebrate *E. crypticus* after exposure to each scenario for 48 h. Solid lines within boxes indicate the median value (*n* = 3). Boxes include data within the 25th and 75th percentiles and whisker lines refer to the 5th and 95th percentiles. Standard refers to the conditions recommended by the standardized OECD guidelines. The symbol * indicates statistical differences compared to standard conditions based on one-way ANOVA followed by Dunnett’s post hoc test (*p* < 0.05). UV (ultraviolet).

The impact of the different scenarios tested on the soil’s bacterial richness and diversity was similar regardless of the absence or presence of the *E. crypticus* (Figure 2). However, this was not the case of the soil incubations performed under drought conditions, where a more significant impact on soil bacterial richness was observed compared to standard conditions when invertebrates were present. A significant increase in bacterial richness (number of OTUs) was observed after exposure to air temperatures ranging between 15 °C and 25 °C, regardless of the absence or presence of the *E. crypticus* (35% and 15%, respectively) (Figure 2a,c). However, when the *E. crypticus* was present in the soil, a decrease in bacterial richness of 21% was also observed under drought conditions (Figure 2c). The bacterial diversity (Shannon–Wiener index) significantly decreased compared to the standard conditions after exposure to air temperatures ranging between 20 °C and 30 °C, under flood and drought scenarios (Figure 2b,d). This occurred regardless of the absence or presence of the *E. crypticus* in the soil (decreases of 7–10% at 20–30 °C, 5–14% under flood and 7% under drought conditions).

**Figure 3:**
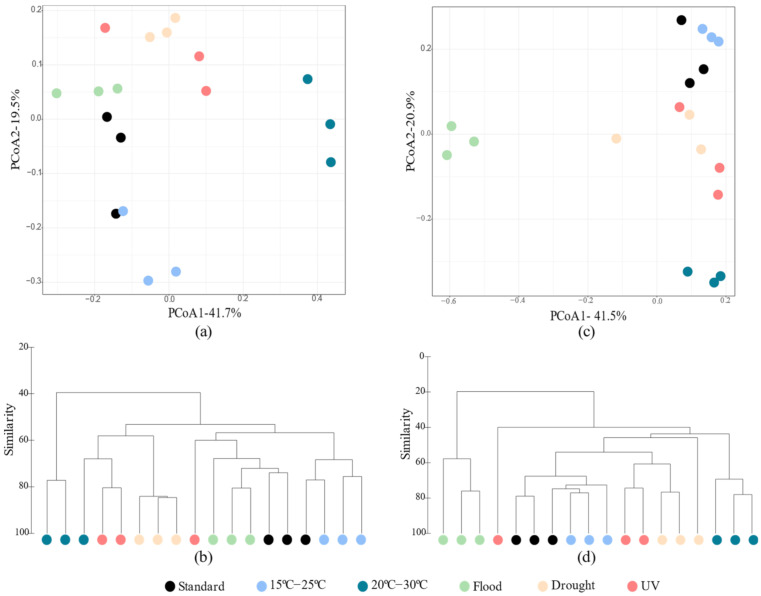
The β diversity measures of Lufa 2.2 soil without (**a**,**b**) and with (**c**,**d**) the presence of the soil invertebrate *E. crypticus* after exposure to each scenario for 48 h. Principal coordinate analyses (PCoAs **a**,**c**) and dendrograms (**b**,**d**) were based on Bray–Curtis similarity matrices of OTUs abundance tables. Standard refers to the conditions recommended by the standardized OECD guidelines. UV (ultraviolet).

The structure of the soil bacterial community was affected differently depending on whether invertebrates were present. The β diversity measures indicated a stronger impact of rising air temperature (20–30 °C) and flood conditions on the soil’s bacterial structure (Figure 3). Without *E. crypticus*, the PCoA revealed a clear separation between the soil bacterial communities exposed to 20–30 °C and those under standard conditions, sharing a similarity of less than 50% (Figure 3a,b). With the presence of the *E. crypticus* in soil, the strongest shift in bacterial structure was observed under flood conditions and temperatures of 20–30 °C (less than 40% and 50% similarity with respect to standard conditions, respectively) (Figure 3c,d).

#### 3.2.2. Taxonomic Composition of Bacterial Communities

The tested scenarios altered the composition of the soil bacterial communities. Effects were observed at class, genera and OTU level, although both the magnitude and the direction of the effects varied among the taxa and depending on the absence or presence of invertebrates in the soil. Additionally, when the *E. crypticus* was present, the effects of flooding were exacerbated. 

**Figure 4:**
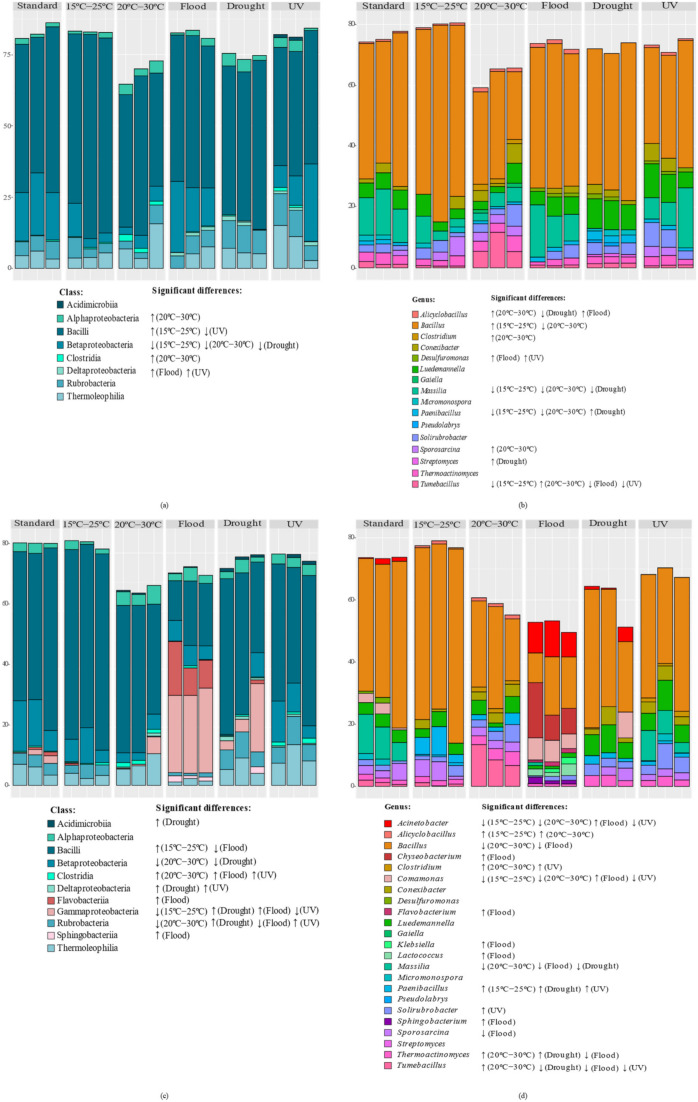
Relative abundance of bacterial classes (**a**,**c**) and 15 most abundant genera (**b**,**d**) of Lufa 2.2 soil without (**a**,**b**) and with (**c**,**d**) the presence of *E. crypticus* after exposure to each scenario for 48 h. Results from three replicates are presented. Standard refers to the conditions recommended by the standardized OECD guidelines. Arrows indicate significant differences (↓ decrease; ↑ increase) compared to standard conditions (one-way ANOVA followed by Dunnett’s post hoc test; *p* < 0.05). UV (ultraviolet).

In the soil without *E. crypticus*, Bacilli and Betaproteobacteria were the dominant bacterial classes under standard conditions (53 ± 4% and 18 ± 2%, respectively) (Figure 4a). Bacilli remained as the most abundant class in the soil after exposure to all the scenarios simulated, although its relative abundance changed significantly under air temperatures ranging between 15 °C and 25 °C (66 ± 5%) and UV radiation (44 ± 2%) (Figure 4a). The relative abundance of Betaproteobacteria also decreased significantly after exposure to rising air temperatures (6 ± 4% at 15–25 °C and 4 ± 1% at 20–30 °C) and were not detected under drought conditions (Figure 4a). In the particular case of exposures to 20–30 °C, Thermoleophilia became the dominant bacterial class in the soil together with Bacilli (Figure 4a). In the absence of *E. crypticus*, the Alphaproteobacteria (increase under temperatures of 20–30 °C), Clostridia (increase under temperatures of 20–30 °C) and Deltaproteobacteria (increase under flood and UV) classes were also significantly affected in relation to the standard conditions (Figure 4a). Deltaproteobacteria, which was not detected under standard conditions, reached a relative abundance of ≈1% after exposure to the flood and UV radiation scenarios.

When the soil was incubated with *E. crypticus*, Bacilli and Betaproteobacteria were still dominant under the standard conditions (55 ± 6% and 14 ± 5%, respectively) (Figure 4c). Exposure to air temperatures ranging between 20 °C and 30 °C induced a significant decrease in the Betaproteobacteria class to 4 ± 1%, while Bacilli (46 ± 6%) and Thermoleophilia (8 ± 2%) became the dominant classes (Figure 4c). The latter were also the dominant bacterial classes in the soil exposed to UV radiation (46 ± 5% for Bacilli and 10 ± 6% for Thermoleophilia) (Figure 4c). After soil exposure to flood conditions, the relative abundance of Bacilli decreased significantly, to 19 ± 4%, although it remained as the dominant class, together with Gammaproteobacteria (28 ± 1% vs. 2 ± 1% under standard conditions) (Figure 4c). Bacilli and Gammaproteobacteria were also the dominant bacterial classes under the drought conditions (45 ± 9% and 10 ± 6%, respectively) (Figure 4c). Compared to the standard conditions, the Gammaproteobacteria class was not detected after exposure to 15–25 °C and UV radiation, while the Rubrobacteria class was not detected after exposure to 20–30 °C (Figure 4c). By contrast, the Acidimicrobiia, Deltaproteobacteria and Sphingobacteriia classes, which were not detected under the standard conditions, reached a relative abundance of ≈1% under drought conditions (Acidimicrobiia); ≈0.5–1% under drought and UV radiation (Deltaproteobacteria); and ≈2% under flood conditions (Sphingobacteriia) (Figure 4c).

At the genus level, regardless of the absence or presence of the *E. crypticus*, all the scenarios simulated significantly affected at least one of the 15 most abundant bacterial genera present in the soil (Figure 4b,d). In the soil without the *E. crypticus*, the exposure to air temperatures ranging between 20 °C and 30 °C led to the strongest shifts in bacterial genera. Compared to the standard conditions, this temperature scenario significantly increased the relative abundances of the *Alicyclobacillus*, *Clostridium*, *Sporosarcina* and *Tumebacillus* genera, while it decreased those of *Bacillus*, *Massilia* and *Paenibacillus* (Figure 4b). The *Clostridium* genus was not detected in the soil under standard conditions. Similarly, exposure to soil moisture alterations and UV radiation led to the detection of the *Desulfuromonas* (flood and UV) and *Streptomyces* (drought) genera, which were not detected under standard conditions (Figure 4b), in the soil. The *Alicyclobacillus*, *Massilia* and *Paenibacillus* genera, which were present in the soil under standard conditions, were not detected after exposure to drought (*Alicyclobacillus* and *Massilia*) and temperatures of 15–25 °C (*Paenibacillus)* (Figure 4b). 

In the presence of *E. crypticus*, exposure to flood conditions was the scenario that caused the strongest changes in the soil bacterial genera. Notably, these conditions induced a significant increase in the *Acinetobacter*, *Chyseobacterium*, *Comomonas*, *Flavobacterium*, *Klebsiella*, *Lactococcus* and *Sphingobacterium* genera, along with a decrease in the *Bacillus*, *Massilia*, *Sporosarcina*, *Thermoactinomyces* and *Tumebacillus* genera (Figure 4d). The *Chyseobacterium*, *Flavobacterium*, *Klebsiella*, *Lactococcus* and *Sphingobacterium* genera were not detected in the soil under standard conditions. By contrast, the *Sporosarcina*, *Massilia* and *Tumebacillus* genera, which were present in the soil under standard conditions, were not detected after exposure to the flood scenario. Similarly, some bacterial genera were also not detected in the soil after exposure to both temperature regimes (*Acinetobacter* and *Comomonas*), drought (*Massilia* and *Tumebacillus*) and UV radiation (*Acinetobacter*, *Comomonas* and *Tumebacillus*) (Figure 4d).

**Figure 5:**
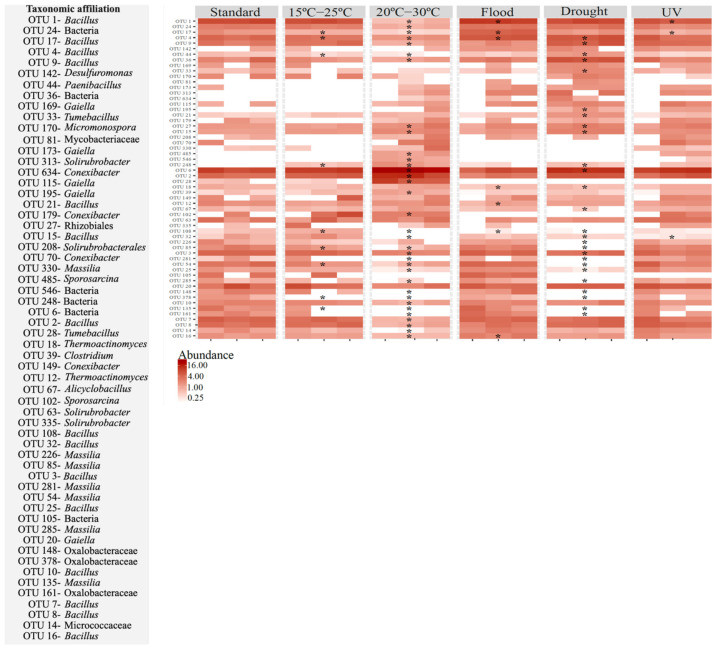
Heatmap for the most heavily represented OTUs (30 most abundant) in Lufa 2.2 soil without the presence of the soil invertebrate *E. crypticus* after exposure to each scenario for 48 h. The color codes represent the relative abundance of OTUs in each soil replicate (*n* = 3). Standard refers to the conditions recommended by the standardized OECD guidelines. The symbol * indicates statistical differences compared to standard conditions based on one-way ANOVA followed by Dunnett’s post hoc test (*p* < 0.05). UV (ultraviolet).

**Figure 6:**
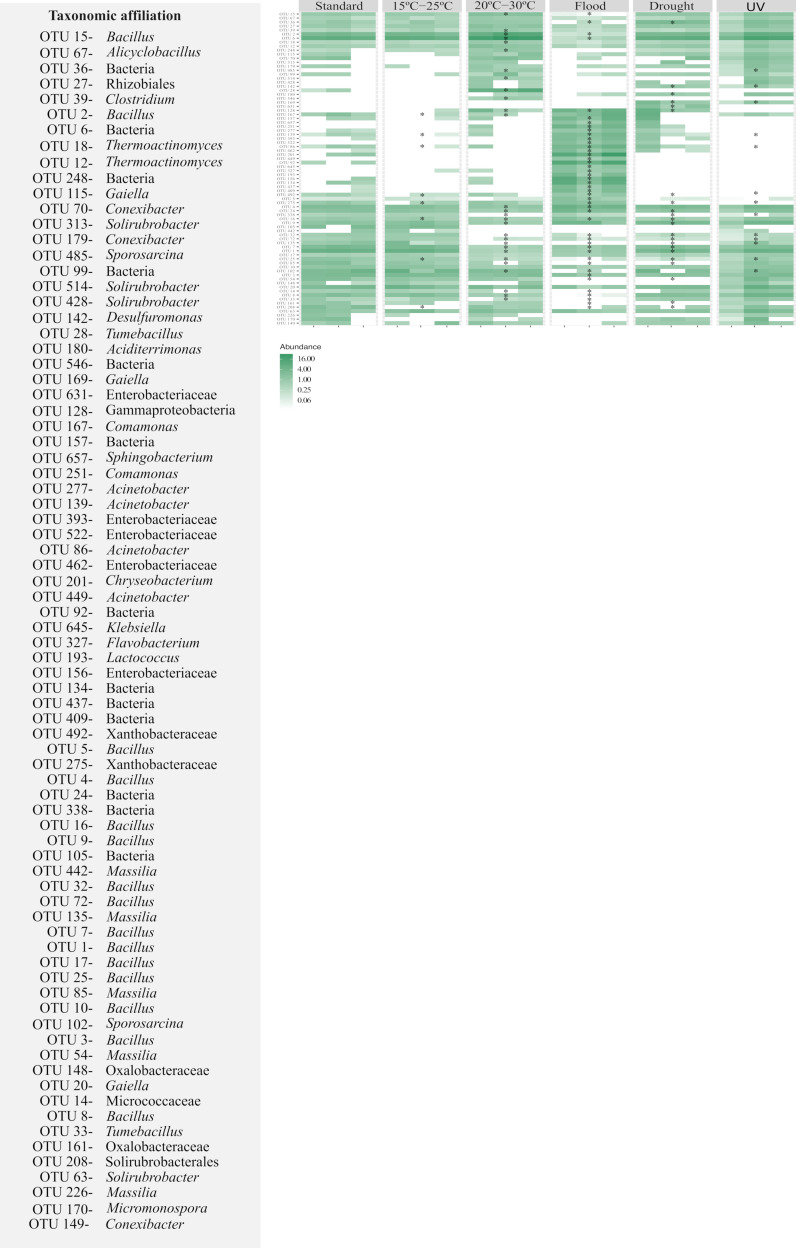
Heatmap for the most heavily represented OTUs (30 most abundant) of Lufa 2.2 soil with the presence of the soil invertebrate *E. crypticus* after exposure to each scenario for 48 h. The color codes represent the relative abundance of OTUs in each soil replicate (*n* = 3). Standard refers to the conditions recommended by the standardized OECD guidelines. The symbol * indicates statistical differences compared to standard conditions based on one-way ANOVA followed by Dunnett’s post hoc test (*p* ≤ 0.05). UV (ultraviolet).

The effect of the different scenarios simulated at the OTU level varied, depending on the absence or presence of soil invertebrates (Figure 5 and Figure 6). In the soil without *E. crypticus*, exposure to the alterations affected 59% (20–30 °C), 45% (drought), 16% (15–25 °C), 12% (flood) and 5% (UV) of the 30 most abundant OTUs in the soil (Figure 5). In the case of air temperatures ranging between 20 °C and 30 °C, 71% of the affected OTUs suffered a decrease in their relative abundance. These OTUs belonged to the *Bacillus*, *Massilia* and *Paenibacillus* genera. Moreover, after exposure to 20–30 °C, 18% of the affected OTUs were not detected in the soil and belonged to the *Bacillus* and *Massilia* genera. Exposure to drought conditions decreased the relative abundance of 54% of the affected OTUs, which belonged to the *Bacillus*, *Massilia*, *Alicyclobacillus* and *Thermoactinomyces* genera. Of the OTUs affected by drought, 35% were not detected after exposure to this scenario and belonged to the *Massilia* genus. 

For soil with *E. crypticus*, the strongest impact resulted after exposure to flood conditions (58% of the top 30 most abundant OTUs affected) (Figure 6). The relative abundance of 60% of the affected OTUs increased after exposure to this scenario. These OTUs belonged to the *Acinetobacter*, *Bacillus*, *Chryseobacterium*, *Comamonas*, *Flavobacterium*, *Klebsiella*, *Lactococcus* and *Sphingobacterium* genera. Furthermore, after exposure to flooding, 13% of the affected OTUs were not detected in the soil; they belonged to the *Massilia* and *Tumebacillus* genera. For this soil, the exposure to 20–30 °C, drought conditions, UV radiation and temperatures of 15–25 °C affected 36%, 28%, 17% and 10% of the OTUs present in the soil, respectively.

### 3.3. Predicted Functional Pathways

The predicted functions depended on the scenarios simulated and, on the presence, or absence of soil invertebrates. These differences were most obvious when the soil was exposed to temperatures between 15 °C and 25 °C. For this scenario, the presence of invertebrates attenuated the impact on the predicted bacterial functions. 

For the soil without the *E. crypticus*, rising air temperatures led to a significant decrease in all the 30 most abundant predicted bacterial functions in the soil (Table S2, Supplementary Material). The biosynthesis of secondary metabolites, carbon metabolism, fatty acid metabolism, carbohydrate metabolism (i.e., glycolysis/gluconeogenesis metabolism, pentose phosphate pathway and citrate cycle), energy metabolism (i.e., oxidative phosphorylation and carbon fixation pathways in prokaryotes), nucleotide metabolism (i.e., purine and pyrimidine metabolism), amino acid metabolism (i.e., glycine, serine and threonine metabolism) and translation (aminoacyl-tRNA biosynthesis and ribosome)-related pathways were some of the functions affected. Although it was not one of the most abundant functions in soil, exposure to rising air temperature increased the metabolism of sulfur. Conditions that mimicked the drought scenario affected 67% of the 30 most abundant predicted bacterial functions in the soil (Table S2, Supplementary Material). In fact, a significant increase in the majority of the affected functions (i.e., biosynthesis of amino acids, carbon metabolism, ABC transporters, carbon fixation pathways and citrate cycle) was observed. Exposure to drought also increased the metabolism of sulfur and nitrogen (which were not among of the 30 most abundant functions in the soil). By contrast, significant decreases were observed in the bacterial functions related to signal transduction (two-component regulatory system), energy metabolism (oxidative phosphorylation)-, cell motility (flagellar assembly and bacterial chemotaxis), carbohydrate metabolism (i.e., glyoxylate and dicarboxylate, amino sugar and nucleotide sugar) and amino acid metabolism (i.e., glycine, serine and threonine) pathways. Exposure to flood conditions significantly decreased the quorum sensing-related pathways (Table S2, Supplementary Material). However, exposure to UV radiation did not affect any of the 30 most heavily represented functions in the soil (Table S2, Supplementary Material).

In the soil with the *E. crypticus*, exposure to flood conditions was the scenario that most affected the 30 most abundant predicted bacterial functions i (40%) (Table S3, Supplementary Material). Exposure to these conditions led to significant decreases in fatty acid metabolism and in the functions related to translation (ribosome), energy metabolism (carbon fixation pathways), cell motility (flagellar assembly), carbohydrate metabolism (i.e., pyruvate, glyoxylate and dicarboxylate) and amino acid metabolism (glycine, serine and threonine) pathways. By contrast, flooding led to significant increases in ABC transport-related functions, the pentose phosphate pathway, the phosphotransferase system, 2-Oxocarboxylic acid and fructose and mannose metabolism. Although it was not one of the most abundant functions in the soil, this scenario also increased the metabolism of nitrogen. Exposure to rising air temperature only affected 17% of the 30 most abundant predicted bacterial functions in the soil (Table S3, Supplementary Material). Significant increases were observed in propanoate metabolism (15–25 °C), fatty acid metabolism and starch and sucrose metabolism (20–30 °C). By contrast, significant decreases were observed in sulfur metabolism (15–25 °C), oxidative phosphorylation and flagellar-assembly pathways (20–30 °C). Exposure to drought conditions and UV radiation affected 14% of the t 30 most heavily represented predicted bacterial functions in the soil (Table S3, Supplementary Material). Drought significantly increased the fatty acid metabolism pathway and decreased methane and sulfur metabolism (energy metabolism related pathways), the pentose phosphate pathway (carbohydrate metabolism) and flagellar assembly. UV radiation exposure significantly decreased the ABC transporter-related functions and methane metabolism and increased the fatty-acid- and carbohydrate-metabolism-related pathways (propanoate and butanoate metabolism).

## 4. Discussion

To understand how soil’s functions and services might be affected by climate change, it is essential to explore how soil microbial communities change in response. Short-term studies can provide important insights into the responses of communities to extreme and occasional events and offer an important complement to long-term observations. We investigated the microbial community response of a standard reference soil widely used in environmental studies (Lufa 2.2 soil) to alterations in air temperature, soil moisture content and UV radiation, with and without the presence of *E. crypticus—*a soil invertebrate species with a key role in terrestrial ecosystems functions and services. Using culture-dependent and -independent analyses, we targeted shifts in microbial catabolic activity, bacterial abundance and bacterial community structure, composition and functions after exposure to these scenarios. 

### 4.1. Effects of Rising Air Temperature

Since different microorganisms have distinct optimal temperatures for growth and activity, the impact of temperature on soil microbial community was expected and has been reported in other studies [5,39,40]. In fact, the microbial activity increased significantly under rising air temperatures (15–25 °C and 20–30 °C) with higher utilization rates of, for example, polymers and carbohydrates. This activity boost may have implications for organic matter decomposition and the amount of carbon dioxide released into the atmosphere [41,42], thus exacerbating climate change. However, this effect may be transient, according to results obtained in studies that applied long-term exposures [10,40,43]. Furthermore, previous studies that also used long-term approaches reported increased growth rates and biomass under rising temperatures [44,45,46]. However, in our study, the number of bacterial cells, estimated from the quantification of the 16S rRNA gene, did not change significantly. Thus, under the short-term exposure tested here, carbon sources may preferentially be used for bacterial acclimation, such as by converting carbon sources into energy, which is needed for lipid synthesis to counteract the effect of temperature on cell-membrane fluidity [5,47]. It should be noted, however, that, as we used a short incubation period, the effects observed here may primarily be the result of changes in the activity of fast-growing microbes. Furthermore, since this method required microbes to grow in Biolog EcoPlates wells, the results were based only a subset of the microbial community (mostly copiotrophs). In addition, other microbes may have been eliminated during sample handling.

The overall bacterial diversity decreased after exposure to increased air temperatures (20–30 °C). These findings are in agreement with those of studies that applied long-term exposure periods [44,48,49], suggesting that these effects are detectable after short-term exposures and may persist over time. For example, Sheik et al. [44] found that the bacterial diversity of grassland soil was markedly lower after a 2-year warming period. In line with this, after an incubation period of 112 d, the results obtained by Lin et al. [49] showed that the bacterial diversity of bamboo soils decreased significantly with increasing temperatures. Reduced diversity limits the contribution of soil microbial communities to terrestrial ecosystem functions and services [50]. This may lead, for example, to a reduction in soil fertility, which decreases the capacity to support plant growth [50].

On the other hand, the scenarios that mimicked an increase in air temperature led to shifts in the soil bacterial community’s structure, which may also have contributed to changes in microbial activity. In fact, of all the scenarios simulated, exposure to an air-temperature regime of between 20 °C and 30 °C was the scenario with the strongest impact on the bacterial community’s structure. The temperature-induced increase in the availability of organic carbon may explain, at least in part, these pronounced shifts [51]. The warmer conditions probably favored the phylogenetic groups adapted to higher temperatures, while having a negative effect on the relative abundance of bacteria with lower growth rates. Accordingly, an increase in the relative abundance of Alphaproteobacteria was observed at 20–30 °C, with possible effects on several metabolic activities (e.g., photosynthesis, carbon dioxide and nitrogen fixation, etc.) [52]. This temperature effect was previously reported by studies that used both short- and long-term approaches (e.g., [51,53,54]) and was related to the copiotrophic nature of Proteobacteria. However, on the other hand, the Betaproteobacteria decreased significantly. This previously reported response of Betaproteobacteria to warming (in both the short- and the long-term) [55,56] is probably related to low-efficiency resource use [57]. The negative impact of this decrease in soil productivity was anticipated, since Betaproteobacteria genera such as *Massilia*, with a significantly lower relative abundance in soils exposed to this scenario, have been described as playing important roles in plant-growth promotion and reducing the amount of nitrates in soils [58]. On the other hand, Thermoleophilia, which is well adapted to higher temperatures [59,60], became one of the dominant classes in the soil after exposure to elevated air temperatures (20–30 °C). Most of the remaining affected phylogenetic groups belonged to the Firmicutes phylum. Among these, an increase in the relative abundance of Clostridia was observed, specifically of spore-forming bacteria of the genus *Clostridium*, as well as other endospore-producing genera of the Bacilli class (e.g., *Sporosarcina*, *Alcyclobacillus*). Several studies, using both shorter and longer incubation periods, reported increases in Firmicute abundance with increasing temperatures [60,61]. In our study, this effect seems to have been genus-specific. For instance, a significant decrease in the relative abundance of *Bacillus* and *Paenibacillus* was observed, despite the adaptation of these bacteria to a wide range of environmental conditions [62]. This decrease may have affected several fundamental functions in soils, such as the nitrogen fixation, solubilization and mineralization of phosphorus and other nutrients [62]. 

In general, our results suggest an attenuation of the effects of temperature on microbial metabolic activity in the presence of *E. crypticus*. In fact, activity was stimulated in the presence of soil invertebrates, both under standard conditions and in the soil exposed to increased air temperatures, while no significant differences were observed between the control and higher-temperature treatments. Invertebrates play an important role in modulating soil porosity and compaction, increasing oxygenation and water availability [63,64], thus benefiting microbial communities. In terms of the structure of the bacterial community, with few exceptions, similar temperature-induced effects were observed in the presence and absence of soil invertebrates, with most of the affected genera being spore-forming bacteria. 

### 4.2. Effects of Flood and Drought Conditions

As a consequence of flooding, soil pores become filled with water, providing anaerobic conditions and, therefore, room for shifts in the soil microbiome [5]. In this soil, microbial activity increased, corroborating the previously reported correlation with soil moisture content [65], which results in an increase in the availability of nutrients [66]. In fact, after exposure to this scenario, a significant increase in membrane-transport-related functions was predicted, such as those of ABC transporters and the phosphotransferase system (PTS), involved in the uptake of nutrients to cells [67] and in carbohydrate translocation and phosphorylation [68]. 

The flood conditions led to a decrease in bacterial diversity, but the community structure was only slightly affected. The few significant compositional effects included an increase in the relative abundance of Deltaproteobacteria (e.g., genus *Desulforomonas*) and in the spore-forming genus *Alcyclobacillus*. The increase in *Desulfuromonas* was probably related to anaerobic conditions caused by the flooding [69]. 

However, the effects of the flooding were exacerbated when the *E. crypticus* was present in soil. The negative effects on the microbial diversity remained, but they were accompanied by significant effects on the relative abundance of six classes and twelve genera. Most of these taxa became more prevalent, namely Gammaproteobacteria, Flavobacteriia and Sphingobacteriia. The abundance of these classes has been positively correlated with soil moisture content, probably due to their copiotrophic lifestyle and, therefore, their prevalence under conditions of higher resource availability [65,66,70]. Among the positively affected genera, *Klebsiella* includes bacteria involved in important soil functions, such as nitrogen fixation, ammonia production, phosphate solubilization and plant-growth promotion [71]. Although, under flood conditions, there is an increase in the availability of nutrients, the anaerobic conditions resulting from excess water may limit the proliferation of bacteria, which probably results in mild effects, as was observed in the soil without soil invertebrates. The presence of *E. crypticus* promotes soil oxygenation and the transport of microorganisms, thus allowing bacteria to benefit from the abundance of nutrients, which may explain the effects observed in the soil with soil invertebrates.

Exposure to the drought scenario led to a decrease in microbial activity, which was in agreement with the results of other studies that used longer exposure times [72]. Several factors may contribute to this decrease, such as, for example, the fact that most of the substrates used by bacteria are water-soluble, or the fact that the scarcity of soil water limits access to these nutrients [73,74]. Drought also had an impact on the bacterial community’s structure and significantly decreased the bacterial diversity, as previously reported by long-term studies [75]. For instance, this scenario led to a decreased relative abundance of Betaproteobacteria, particularly the *Massilia* genus. As described previously, this result could be concerning due to the important functions carried out by this genus in soil [58]. On the other hand, when used as part of a strategy to deal with drought, some bacteria are able to form endospores, allowing their survival in a dormant state [76]. This was the case of some of the genera whose relative abundance increased significantly in this scenario, such as *Paenibacillus* and *Streptomyces*. One of the predicted functions that was significantly affected was fatty acid metabolism, which was projected to be higher in the drought-treated soils. This result may have been related to the microbial response to drought, which involves altering the lipids content in cell membranes [77]. Fatty acids can also be used as alternative carbon sources when nutrients are scarce [78].

In the presence of the *E. crypticus*, similar effects were observed in terms of the microbial activity, diversity and community structure. However, a positive effect was observed on the relative abundance of Gammaproteobacteria, Deltaproteobacteria, Acidimicrobiia and Rubrobacteria. While the negative correlation of Rubrobacteria and Acidimicrobiia to soil moisture has been reported [66], the opposite result was expected for the Proteobacteria due to their sensitivity to desiccation [79]. Additionally, the number of predicted functions significantly affected was considerably lower in the presence of soil invertebrates (14% vs. 67%). As stated above, drought may result in soil dissection, limiting microbial access to water and nutrients. Under these conditions, soil invertebrates may benefit the microbiome by breaking up soil aggregates and facilitating microbial dispersion.

### 4.3. Effects of UV Radiation Exposure

Of the scenarios tested, the increase in UV radiation was the one that least affected the microbial community. For example, the microbial diversity was not affected, probably due to the resistance of the soil-surface bacteria to the UV radiation [80,81]. Nevertheless, in the absence of soil invertebrates, exposure to UV radiation increased the microbial activity and affected the relative abundance of Bacilli (e.g., a significant decrease in *Tumebacillus* and OTUs affiliated with *Bacillus*) and Deltaproteobacteria (e.g., significant increase of *Desulfuromonas*). As mentioned previously, decreases in *Bacillus* may affect the important functions carried out by this bacterium (i.e., nitrogen fixation and solubilization and mineralization of phosphorus and other nutrients) [61]. Alterations in bacterial communities after exposure to UV radiation were reported previously [81,82] and these effects may be related to changes in soil properties. Johnson et al. [82], for example, reported an effect of UV on the carbon-to-nitrogen ratio. 

In the presence of the *E. crypticus,* no effects on the microbial activity were observed. However, exposure to this scenario led to changes in several of the predicted functions for this soil bacterial community, such as the decrease in methane metabolism. This could have been related to the reduced methane efflux in the soil with the increase in UV radiation [83,84]. Furthermore, the phylogenetic groups affected by the UV radiation were different in the presence of soil invertebrates, once again emphasizing the importance of evaluating the effects of climate factors on the microbiome by considering interactions with other organisms.

## 5. Conclusions

Our study demonstrated that short-term alterations in air temperature, soil moisture content and UV radiation have significant consequences for the activity, composition, structure and function of soil microbial communities. All the scenarios simulated had an impact on the microbial activity and bacterial community’s composition. Additionally, except for UV radiation, all the conditions tested led to a decrease in bacterial diversity. Our results also indicated that different taxa respond differently to the same scenario. The overall microbial community structure was particularly affected by increases in air temperature (20–30 °C, in the absence of *E. crypticus*) and flood conditions (in the presence of *E. crypticus*). The presence of soil invertebrates changed the microbial community response to the scenarios tested, mostly resulting in an attenuation of the observed effects. These results highlight the importance of using realistic scenarios when assessing the effects of climate change on the soil microbiome, including the consideration of the interaction of soil microbial communities with other biotic factors, such as the presence of soil invertebrates.

In the future, climate change will shape the way ecosystems function, with significative effects on the functions of ecosystems services, such as soil fertility and productivity, which could lead to negative environmental, social and economic effects. The soil microbiome is fundamental for these functions. More studies under realistic scenarios are needed in order to use this knowledge to try to mitigate the negative effects of climate change on soils.

## Supplementary Materials

The following supporting information can be downloaded at: https://www.mdpi.com/article/10.3390/genes13050850/s1. Figure S1: Visual representation of the scenarios simulated: (a) air temperature; (b) soil moisture content; (c) ultraviolet (UV) radiation. Figure S2: Experimental set-up for (UV) radiation scenario. Figure S3: Bacterial absolute abundance with and without *E. crypticus* after exposure to each scenario tested. Table S1: Summary of the scenarios simulated. Table S2: Predicted functional pathway changes without *E. crypticus* after exposure to each scenario tested. Table S3: Predicted functional pathway changes with *E. crypticus* after exposure to each scenario tested.
