# Supplementary material for: Short-Term Responses of Soil Microbial Communities to Changes in Air Temperature, Soil Moisture and UV Radiation
Source: Genes (Basel). 2022 May 10;13(5):850. doi: 10.3390/genes13050850 (PMC9142034; doi:10.3390/genes13050850)
Supplement: Supplementary file 1 [file genes-13-00850-s001.zip › genes-1631493-supplementary.pdf]

Supplementary information to:

Short-Term Responses of Soil Microbial Communities to Changes in Air Temperature, Soil Moisture and UV Radiation

Isabel Silva [1,2], Marta Alves [3], Catarina Malheiro [2], Ana Rita R. Silva[2], Susana Loureiro [2], Isabel Henriques [1,*] and M. Nazaret González-Alcaraz [4,*]

[1] CEF (Center for Functional Ecology), Department of Life Sciences, Faculty of Sciences and Technology, University of Coimbra, 3000-456 Coimbra, Portugal; silva.isabel@ua.pt
[2] CESAM (Centre for Marine and Environmental Studies), Department of Biology, University of Aveiro, 3810-193 Aveiro, Portugal; catarinalemosmalheiro@ua.pt (C.M.); ritas@ua.pt (A.R.R.S.); sloureiro@ua.pt (S.L.)
[3] CBQF—Center for Biotechnology and Fine Chemistry, School of Biotechnology, Portuguese Catholic University, 4169-005 Porto, Portugal; msalves@ucp.pt
[4] Department of Agricultural Engineering of the E.T.S.I.A. & Soil Ecology and Biotechnology Unit of the Institute of Plant Biotechnology, Technical University of Cartagena, 30203 Cartagena, Spain
* Correspondence: isabel.henriques@uc.pt (I.H.); nazaret.gonzalez@upct.es (M.N.G.-A.)

* Correspondence: isabel.henriques@uc.pt; nazaret.gonzalez@upct.es

Supplementary material summary: 1 cover page, 1 pages of text, 3 figures and 3 tables:

- Table S1. Summary of the scenarios simulated;
- Table S2. Predicted functional pathway changes without *E. crypticus* after exposure to each scenario tested;
- Table S3. Predicted functional pathway changes with *E. crypticus* after exposure to each scenario tested;
- Figure S1. Visual representation of the scenarios simulated: a) air temperature; b) soil moisture content c) ultraviolet (UV) radiation;
- Figure S2. Experimental set-up for (UV) radiation scenario;
- Figure S3. Bacterial absolute abundance with and without *E. crypticus* after exposure to each scenario tested;

Table S1. Scenarios simulated during the 48 h incubation of soil samples without and with the presence of the soil invertebrate *E. crypticus*. Standard refers to the climate conditions recommended by the standardized OECD guidelines. WHC (water holding capacity). UV (ultraviolet).

**Climate factor**	**Scenario**		
	**Air temperature**	**Soil moisture**	**UV radiation**
Standard	20°C	50% WHC	Without
Air temperature	15°C–25°C	50% WHC	Without
	20°C–30°C	50% WHC	Without
Soil moisture	20°C	75% WHC	Without
	20°C	25% WHC	Without
UV radiation	20°C	50% WHC	With

Table S2. Predicted functional pathway changes (based on top 30 KEGG functions) of Lufa 2.2 soil without the presence of the soil invertebrate *E. crypticus* after exposure to each scenario for 48 h. Only significant differences in relative abundance towards the standard conditions (↓ decrease; ↑ increase) are indicated (one-way ANOVA followed by Dunnett's post-hoc test; $p < 0.05$).

**Climate scenario**	**KEGG pathway**	**KEGG ID**	**Tendency**	***P***
**15°C–25°C and 20°C–30°C**	Metabolic pathways	ko01100	↓	0.05
	Biosynthesis of secondary metabolites	ko01110	↓	0.05
	Microbial metabolism in diverse environments	ko01120	↓	0.00
	Biosynthesis of antibiotics	ko01130	↓	0.05
	Biosynthesis of amino acids	ko01230	↓	0.00
	Aminoacyl-tRNA biosynthesis	ko00970	↓	0.05
	Carbon metabolism	ko01200	↓	0.05
	ABC transporters	ko02010	↓	0.00
	Two-component system	ko02020	↓	0.00
	Ribosome	ko03010	↓	0.00
	Purine metabolism	ko00230	↓	0.05
	Quorum sensing	ko02024	↓	0.05
	Oxidative phosphorylation	ko00190	↓	0.05
	Pyrimidine metabolism	ko00240	↓	0.00
	Pyruvate metabolism	ko00620	↓	0.05
	Glyoxylate and dicarboxylate metabolism	ko00630	↓	0.00
	Glycolysis / Gluconeogenesis	ko00010	↓	0.00
	Flagellar assembly	ko02040	↓	0.00
	Amino sugar and nucleotide sugar metabolism	ko00520	↓	0.00

	Glycine, serine and threonine metabolism	ko00260	↓	0.00
	Cysteine and methionine metabolism	ko00270	↓	0.00
	Propanoate metabolism	ko00640	↓	0.05
	Butanoate metabolism	ko00650	↓	0.00
	Porphyrin and chlorophyll metabolism	ko00860	↓	0.05
	Carbon fixation pathways in prokaryotes	ko00720	↓	0.05
	Fatty acid metabolism	ko01212	↓	0.00
	Alanine, aspartate and glutamate metabolism	ko00250	↓	0.00
	Starch and sucrose metabolism	ko00500	↓	0.05
	Bacterial chemotaxis	ko02030	↓	0.00
	Citrate cycle (TCA cycle)	ko00020	↓	0.05
	Valine, leucine and isoleucine degradation	ko00280	↓	0.00
	Pentose phosphate pathway	ko00030	↓	0.00
	2-Oxocarboxylic acid metabolism	ko01210	↓	0.00
**Drought**	Biosynthesis of secondary metabolites	ko01110	↑	0.05
	Biosynthesis of antibiotics	ko01130	↑	0.05
	Biosynthesis of amino acids	ko01230	↑	0.02
	Carbon metabolism	ko01200	↑	0.05
	ABC transporters	ko02010	↑	0.01
	Two-component system	ko02020	↓	0.01
	Oxidative phosphorylation	ko00190	↓	0.05
	Glyoxylate and dicarboxylate metabolism	ko00630	↓	0.01
	Flagellar assembly	ko02040	↓	0.00
	Amino sugar and nucleotide sugar metabolism	ko00520	↓	0.00
	Glycine, serine and threonine metabolism	ko00260	↓	0.04
	Propanoate metabolism	ko00640	↑	0.05
	Butanoate metabolism	ko00650	↑	0.01
	Porphyrin and chlorophyll metabolism	ko00860	↑	0.05
	Carbon fixation pathways in prokaryotes	ko00720	↑	0.05
	Fatty acid metabolism	ko01212	↑	0.00
	Alanine, aspartate and glutamate metabolism	ko00250	↑	0.00
	Starch and sucrose metabolism	ko00500	↑	0.05
	Bacterial chemotaxis	ko02030	↓	0.02
	Citrate cycle (TCA cycle)	ko00020	↑	0.05
	Valine, leucine and isoleucine degradation	ko00280	↑	0.01
	2-Oxocarboxylic acid metabolism	ko01210	↑	0.01
**Flood**	Quorum sensing	ko02024	↓	0.05

Table S3. Predicted functional pathway changes (based on top 30 KEGG functions) of Lufa 2.2 soil with the presence of the soil invertebrate *E. crypticus* after exposure to each scenario for 48 h. Only significant differences in relative abundance towards the standard conditions (↓ decrease; ↑ increase) are indicated (one-way ANOVA followed by Dunnett's post-hoc test; $p < 0.05$; ANOVA). UV (ultraviolet).

Climate scenario	KEGG pathway	KEGG ID	Tendency	*P*
**15°C–25°C**	Propanoate metabolism	ko00640	↑	0.05
	Sulfur metabolism	ko00920	↓	0.05
**20°C–30°C**	Oxidative phosphorylation	ko00190	↓	0.00
	Flagellar assembly	ko02040	↓	0.05
	Fatty acid metabolism	ko01212	↑	0.05
	Starch and sucrose metabolism	ko00500	↑	0.02
**Drought**	Flagellar assembly	ko02040	↓	0.05
	Fatty acid metabolism	ko01212	↑	0.05
	Methane metabolism	ko00680	↓	0.00
	Pentose phosphate pathway	ko00030	↓	0.04
	Sulfur metabolism	ko00920	↓	0.05
**Flood**	ABC transporters	ko02010	↑	0.00
	Ribosome	ko03010	↓	0.05
	Pyruvate metabolism	ko00620	↓	0.02
	Glyoxylate and dicarboxylate metabolism	ko00630	↓	0.05
	Glycine, serine and threonine metabolism	ko00260	↓	0.03
	Propanoate metabolism	ko00640	↓	0.05
	Carbon fixation pathways in prokaryotes	ko00720	↓	0.03
	Flagellar assembly	ko02040	↓	0.05
	Fatty acid metabolism	ko01212	↓	0.05
	Pentose phosphate pathway	ko00030	↑	0.00
	Starch and sucrose metabolism	ko00500	↑	0.02
	Phosphotransferase system (PTS)	ko02060	↑	0.00
	2-Oxocarboxylic acid metabolism	ko01210	↑	0.00
	Fructose and mannose metabolism	ko00051	↑	0.00
**UV**	ABC transporters	ko02010	↓	0.02
	Propanoate metabolism	ko00640	↑	0.05
	Butanoate metabolism	ko00650	↑	0.03
	Fatty acid metabolism	ko01212	↑	0.05
	Methane metabolism	ko00680	↓	0.02

Description of the experimental set-up for the simulation of the ultraviolet (UV) radiation scenario.

UV radiation was supplied by two UV lamps (peak emissions at 365 nm for UVA and 312 nm for UVB; Spectroline XX15F/B, Spectronics Corporation, USA;) installed inside an acclimatized room in a permanent, static position respect to fixed platforms where the test jars were located. Both UV lamps were covered by cellulose acetate sheets previously burned under the lamps for 12 h. This allowed the stabilization of the UV radiation intensity passing through and prevented the exposure of the test jars to UVC, which is not typically found in nature due to its strong adsorption to the stratosphere ozone layer. One UV lamp was positioned at 75 cm distance from the test jars to reach the UV index of 7 and the other lamp at 65 cm distance to reach the UV index of 10. The test jars were moved between platforms to be exposed to the corresponding UV indexes (perforated parafilm covers were removed from the test jars during the periods of UV radiation emission). The UV radiation intensity reaching the test jars was checked with a spectroradiometer connected to a monochromator (Bentham Instruments, UK). The experimental set-up for the simulation of the UV radiation scenario is shown in Figure S2.

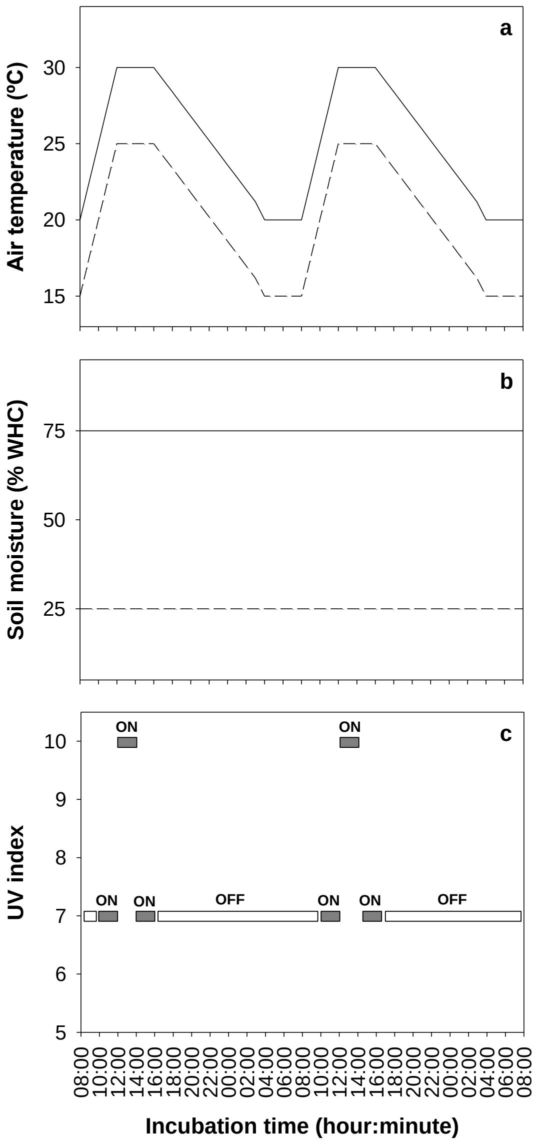

Figure S1. Scenarios simulated during the 48 h incubation of soil samples without and with the presence of the soil invertebrate *Enchytraeus crypticus*: a) air temperature (15°C─25°C and 20°C─30°C); b) soil moisture content (75% and 25% of soil water holding capacity, WHC); c) ultraviolet (UV) radiation (with UV radiation emission). In the UV radiation scenario "ON" indicates that the UV lamps were emitting UV radiation and "OFF" that there was no UV radiation emission.

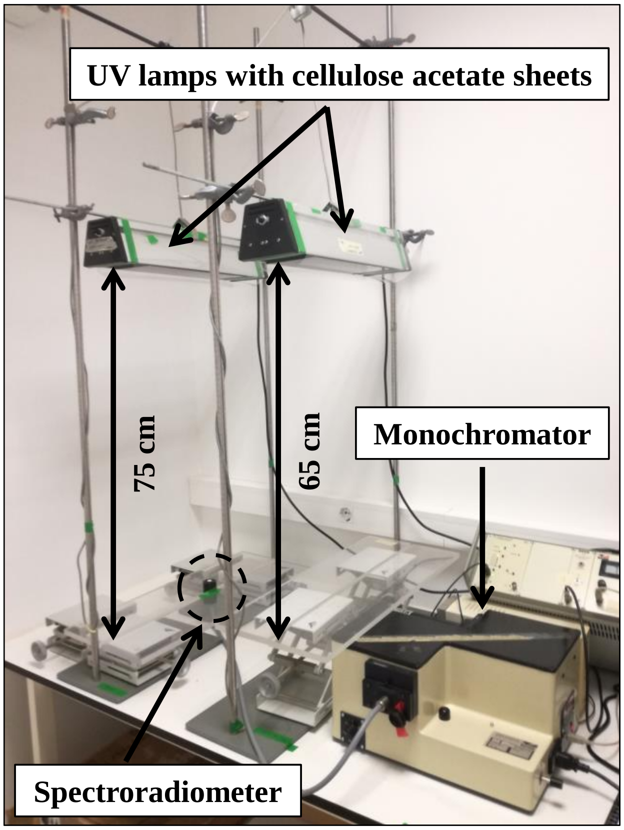

Figure S2. Experimental set-up for the simulation of the ultraviolet (UV) radiation scenario.

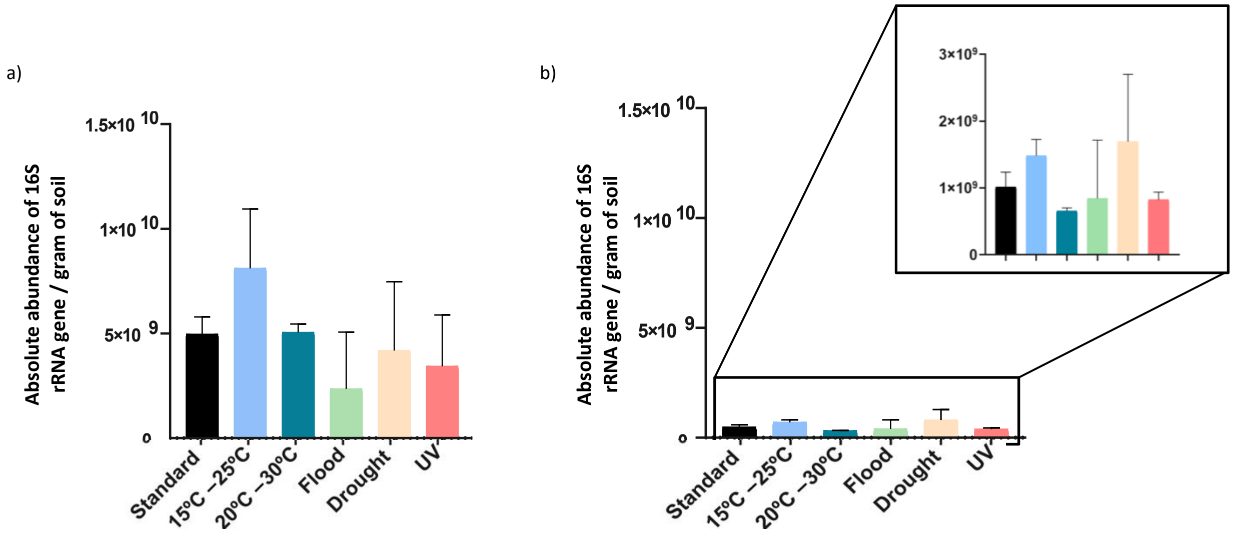

Figure S3. Lufa 2.2 soil without (a) and with (b) the presence of the soil invertebrate *Enchytraeus crypticus* after exposure to each scenario for 48 h. Standard refers to the climate conditions recommended by the standardized OECD guidelines. Values are average ± SD (n=3). UV (ultraviolet).
